# Ubiquitination-mediated PRDX2 alleviates intervertebral disc degeneration via restraining TBHP-induced nucleus pulposus cell apoptosis, ferroptosis and ECM degradation

**DOI:** 10.1186/s12950-026-00493-x

**Published:** 2026-03-24

**Authors:** Fanguo Kong, Jiyao Luan, Qipeng Pan, Yang Qiao, Wenju Wang

**Affiliations:** 1 Department of Spinal Minimally Invasive Surgery II, Henan Luoyang Orthopedic Hospital (Henan Provincial Orthopedic Hospital), No. 100 Yongping Road, Zhengdong New District, Zhengzhou, Henan 450000 China; 2 Department of Interventional, Henan Luoyang Orthopedic Hospital (Henan Provincial Orthopedic Hospital), Zhengzhou, Henan 450000 China

**Keywords:** Intervertebral disc degeneration, Nucleus pulposus, Deubiquitination

## Abstract

**Background:**

Peroxiredogenase 2 (PRDX2) has been confirmed to be downregulated in patients with intervertebral disc degeneration (IDD). However, the role and mechanism of PRDX2 in the progression of IDD are still unclear.

**Methods:**

Tert-butyl hydroperoxide (TBHP)-induced nucleus pulposus (NP) cells were used to construct IDD conditions. qRT-PCR or western blot was used to detect the expression levels of PRDX2, ubiquitin-specific protease 11 (USP11), ferroptosis-related markers and extracellular matrix (ECM)-related proteins. CCK8 assay, TUNEL assay and detection of ferroptosis-related markers were performed to examine cell viability, apoptosis and ferroptosis. Co-IP assay and ubiquitination assay were used to explore the regulation of USP11 on PRDX2.

**Results:**

The expression levels of PRDX2 and USP11 were reduced in IDD patients and TBHP-induced NP cells. PRDX2 overexpression repressed TBHP-induced NP cell apoptosis, ferroptosis and ECM degradation. Moreover, our study found that USP11 stabilized the protein expression of PRDX2 by decreasing its ubiquitination. In addition, USP11 overexpression inhibited TBHP-induced NP cell apoptosis, ferroptosis and ECM degradation, while these effects were reversed by PRDX2 knockdown.

**Conclusion:**

USP11-mediated deubiquitination of PRDX2 might alleviate IDD progression by inhibiting TBHP-induced NP cell apoptosis, ferroptosis and ECM degradation, providing a potential intervention target worthy of further research for IDD.

**Supplementary Information:**

The online version contains supplementary material available at 10.1186/s12950-026-00493-x.

## Introduction

Intervertebral disc degeneration (IDD) is a common musculoskeletal degenerative disease that is considered to be the main cause of low back pain and disability [1, 2]. Degeneration of nucleus pulposus (NP) in the gelatinous central part of the intervertebral disc is an important mechanism of IDD [3, 4]. Several studies have shown that tert-butyl hydroperoxide (TBHP) induces apoptosis, ferroptosis, and extracellular matrix (ECM) degradation in NP cells, which can be used to mimic in vitro IDD models [5]. Therefore, elucidating the underlying molecular mechanisms of TBHP-induced nucleus pulposus cell apoptosis, ferroptosis, and ECM degradation is of great significance for further understanding the pathogenesis of IDD and exploring effective intervention strategies.

Peroxiredoxin-2 (PRDX2) is part of the PRDXs family of antioxidant enzymes, which plays a role in defending against oxidative stress and protecting cells from hydroperoxides [6]. Previous study suggested that PRDX2 overexpression decreased apoptosis, fibrosis and inflammation to ameliorate cardiac diastolic dysfunction [7]. Moreover, silencing of PRDX2 could induce ferroptosis in hepatic stellate cells to alleviate hepatic fibrosis [8]. Besides, knockdown of PRDX2 was confirmed to enhance ECM degradation in chondrocytes [9]. The above reports confirmed the anti-apoptotic, anti-ferroptosis and anti-ECM degradation effects of PRDX2. Tu et al. analyzed the differentially expressed genes in degenerated NP tissues, and found that PRDX2 was lower expressed in severely degenerated NP tissues from IDD patients [10]. However, whether PRDX2 regulates NP cell apoptosis, ferroptosis, and ECM degradation to mediate IDD progression remains unclear.

The ubiquitin-specific protease (USP) family is the largest subclass of deubiquitinating enzymes. Emerging evidence suggests that several USPs, such as USP7 [11] and USP14 [12], play important roles in the pathophysiology of IDD. These studies highlight that specific USPs can exert protective or detrimental effects on intervertebral disc homeostasis, suggesting that targeting the ubiquitination/deubiquitination regulatory network has therapeutic potential for IDD. As a member of this family, USP11 has been implicated in regulating DNA damage repair, cell apoptosis, and tumorigenesis [13–15]. It had been reported that USP11 deubiquitinated and stabilized HIF-1α, thereby accelerating cell glycolysis in hepatocellular carcinoma [16]. USP11 could stabilize LSH via deubiquitination, thus suppressing the ferroptosis of colorectal cancer cells [17]. Previous studies had shown that deubiquitinase USP11 relieved TBHP-induced NP cell ferroptosis by deubiquitinating and stabilizing Sirt3, thereby improving IDD progression [18]. Here, our study detected that USP11 could promote PRDX2 expression. However, whether USP11 regulates PRDX2 level to affect IDD process has not been explored.

This study hypothesized that PRDX2, regulated by USP11-mediated deubiquitination, relieved TBHP-induced NP cell apoptosis, ferroptosis, and ECM degradation to alleviate IDD progression.

## Materials and methods

### Samples collection

All participants were recruited from Henan Luoyang Orthopedic Hospital (Henan Provincial Orthopedic Hospital). Degenerative NP tissues were collected from patients with IDD (*n* = 30) who underwent discectomy surgery for severe discogenic low back pain or radicular symptoms. Normal NP tissues were obtained from patients (*n* = 28) who required vertebral resection and internal fixation for acute traumatic fractures, but whose intervertebral discs were confirmed to be normal by visual observation during surgery. The study was approved by the Ethics Committee of Henan Luoyang Orthopedic Hospital (Henan Provincial Orthopedic Hospital), and written informed consent was obtained from each participant. This study was carried out in strict accordance with the relevant regulations and requirements of the Declaration of Helsinki.

### Cell culture, treatment and transfection

Human intervertebral disc NP cells (CP-H097, Procell, Wuhan, China) were cultured in specific completed medium (CM-H097, Procell). To simulate IDD conditions, NP cells were exposed to 0, 25, 50 and 100 µM of TBHP for 3 h. NP cells were transfected with PRDX2/USP11 overexpression vectors, small interfering RNAs (siRNAs) against PRDX2/USP11 (si-PRDX2: F 5’-UGUGUUUGGAGAAAUAUUCCU-3’, R 5’-GAAUAUUUCUCCAAACACAAU-3’; si-USP11: F 5’-UUAUCUCAUCUUGAAAGAGUG-3’, R 5’-CUCUUUCAAGAUGAGAUAAAC-3’) or siRNA negative control (si-NC: F 5’-GGAGUAGGGAGCAAACCUAUAGGAA-3’, R 5’-UUCCUAUAGGUUUGCUCCCUACUCC-3’), synthesized by RiboBio (Guangzhou, China), using Lipofectamine 3000 (Invitrogen, Carlsbad, CA, USA). The PRDX2 or USP11 overexpression vectors were constructed by inserting the PCR products of PRDX2 or USP11 into pcDNA3.1 vectors. After 48 h, NP cells were treated with 100 µM TBHP for 3 h as previously described [18].

### qRT-PCR

Total RNA was extracted by RNA simple (Tiangen, Beijing, China) and reverse-transcribed into cDNA with PrimeScript RT Reagent Kit (Takara, Tokyo, Japan). Subsequently, cDNA was mixed with SYBR Green (Takara) and specific primers (Table 1) to perform qRT-PCR. Relative PRDX2 and USP11 levels were tested by 2^−ΔΔCt^ method, with GAPDH as housekeeping gene.

**Table 1:** Primer sequences used for qRT-PCR

Name		Primers for PCR (5’-3’)
PRDX2	Forward	CCACCTGGCTTGGATCAACA
	Reverse	TTTCAGCACGCCGTAATCCT
USP11	Forward	TTCCACGGCCTCTTCAAGTC
	Reverse	CGCGGATCCATGGGGATAAA
GAPDH	Forward	CAAATTCCATGGCACCGTCA
	Reverse	GACTCCACGACGTACTCAGC

### Western blot (WB)

Three cases of degenerative tissues and three cases of normal control tissues were randomly selected from the sample bank for WB analysis. Total proteins were extracted from tissues and cells by RIPA buffer, separated by 10% SDS-PAGE electrophoresis and transferred into PVDF membranes. Then, membrane was incubated with anti-GAPDH (1:50000, 60004-1-Ig, Proteintech, Rosemont, IL, USA), anti-PRDX2 (1:4000, 10545-2-AP, Proteintech), anti-USP11 (1:600, 10244-1-AP, Proteintech), anti-ACSL4 (1:10000, ab155282, Abcam, Cambridge, CA, USA), anti-GPX4 (1:1000, ab125066, Abcam), anti-Aggrecan (1:1000, ab3778, Abcam), anti-COL2A1 (1:5000, ab188570, Abcam), anti-Ub (1:10000, ab134953, Abcam), Goat anti-Rabbit (1:50000, ab205718), or Goat anti-mouse IgG (1:10000, ab205719, Abcam). Protein signals were detected after ECL reagent (Beyotime, Shanghai, China) treatment. Gray value was analyzed by ImageJ software with GAPDH as loading control. The full length original WB images are shown as supplemental files.

### CCK8 assay

NP cells were seeded into 96-well plates (2 × 10^3^ cells/well) and cultured for 48 h. After that, cells were treated with 10 µL CCK8 reagent (Beyotime) for 2 h. Optical density (OD) value was analyzed at 450 nm using a microplate reader, and cell viability (%) was calculated by OD_treatment group_/OD_control group_×100%.

### Cell cytotoxicity detection

LDH Cytotoxicity Assay Kit (C0016, Beyotime) was used to evaluate LDH level. Briefly, NP cells were cultured until 90% confluency in 96-well plates (3 × 10^4^ cells/well). After centrifugation (400 g for 5 min), cells were incubated with LDH release reagent for 1 h. Then, cells were centrifuged, and cell supernatant was collected and incubated with LDH test working solution for 30 min. LDH level was evaluated to assess cell cytotoxicity under a microplate reader at 490 nm.

### TUNEL staining

NP cells in 96-well plates (1 × 10^4^ cells/well) were fixed with 4% paraformaldehyde, treated with 0.3% Triton X-100, and incubated with TUNEL solution. After stained with DAPI solution, TUNEL-positive cells in five randomly selected visual fields were observed under a fluorescence microscope (100×) and analyzed by ImageJ software.

### Cell ferroptosis detection

The ROS, MDA, GSH, and Fe^2+^ levels in NP cells were detected by DCFDA/H2DCFDA Cellular ROS Assay Kit (ab113851, Abcam), MDA Assay Kit (ab118970, Abcam), GSH Assay Kit (ab239727, Abcam), and Iron Assay Kit (ab83366, Abcam), respectively.

### Cycloheximide (CHX) treatment

NP cells transfected with si-NC/si-USP11 were treated with 20 µg/mL CHX solution. At each time point (0, 2, 4 and 8 h), cells were collected for detecting USP11 and PRDX2 protein levels using WB.

### Co-IP assay

To confirm the interaction between PRDX2 and USP11, Co-IP assay was performed. Briefly, NP cells were lysed with IP lysis buffer containing 50 mM Tris-HCl, 150 mM NaCl, 10 mM KCl, 0.5% NP-40, 1 mM EDTA, 1.5 mM MgCl_2_, 10% glycerol, and protease inhibitor. After that, cell lysates were treated with anti-USP11, anti-PRDX2 or anti-IgG in TBS buffer, and then hatched with protein A/G agarose beads (Beyotime) overnight at 4 °C on a vertical roller. Proteins were eluted and used for WB to detect PRDX2 and USP11 protein levels.

### Ubiquitination assay

To detect the effect of USP11 knockdown on PRDX2 ubiquitination, NP cells were transfected with HA-UB, si-NC/si-USP11 for 48 h, and then cell lysates were hatched with anti-HA or anti-PRDX2 and treated with protein A/G agarose beads. Samples were boiled for WB assay.

NP cells were transfected with plasmid expressing HA-tagged ubiquitin (K48-linked-UB and K63-linked-UB) and si-NC/si-USP11. After lysis, cells lysates were immunoblotted with anti-HA using protein A/G agarose beads. The ubiquitination level of PRDX2 was analyzed by WB.

### Statistical analysis

All experiments were performed in triplicate, with each independent experiment set 3 times to generate an average value. All data were analyzed by GraphPad Prism 8.0 software and shown as mean ± SD. Different between groups were compared using Student’s *t*-test or ANOVA. *P* < 0.05 was considered as statistically significance.

## Results

### PRDX2 was downregulated in IDD patients and TBHP-induced NP cells

**Fig. 1:**
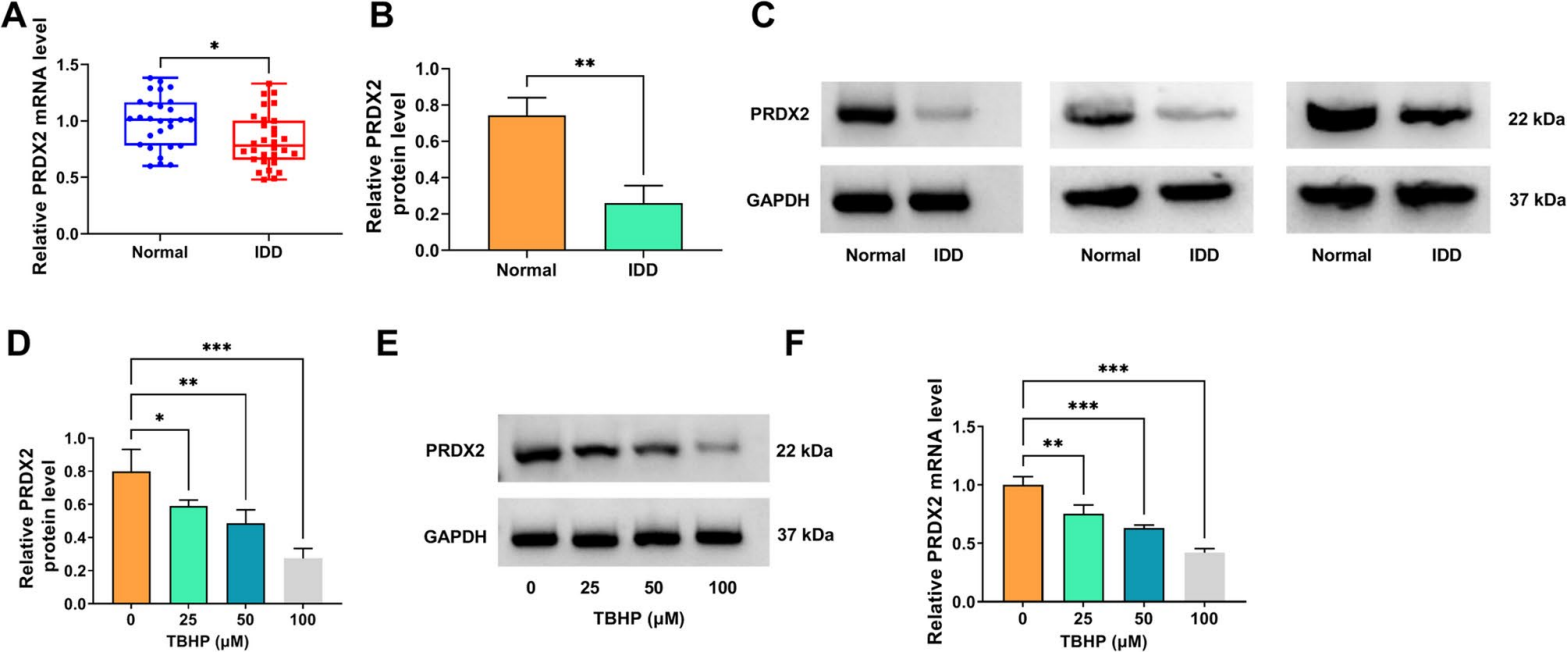
PRDX2 expression in IDD patients and TBHP-induced NP cells. (**A**) PRDX2 mRNA level was detected by qRT-PCR in the NP tissues of IDD patients (*n* = 30) and normal controls (*n* = 28). The dots represent individual patient samples. (**B-C**) PRDX2 protein level in the NP tissues of IDD patients and normal controls was measured by WB. Data were derived from 3 independent human tissue samples (*n* = 3). (**D-F**) PRDX2 mRNA and protein levels were examined by qRT-PCR and WB in NP cells treated with different concentrations of TBHP for 3 h (*n* = 3). **A** and **B**, Student’s *t*-test; **D** and **F**, one-way ANOVA. *P < 0.05, **P < 0.01, ***P < 0.001

Firstly, our study determined PRDX2 expression in IDD patients and TBHP-induced cells models. Compared with normal controls, PRDX2 was lower expressed in the NP tissues of IDD patients at the mRNA and protein levels (Fig. 1 A-C). In TBHP-induced NP cells, PRDX2 mRNA and protein expression was gradually decreased with increasing concentration of TBHP treatment (Fig. 1 D-F). The observed dose-dependent downregulation of PRDX2 under TBHP-induced oxidative stress suggests that PRDX2 is not merely a bystander but a potential target compromised during IDD-associated oxidative damage, prompting us to investigate its functional role.

### PRDX2 overexpression suppressed TBHP-induced NP cell apoptosis

**Fig. 2:**
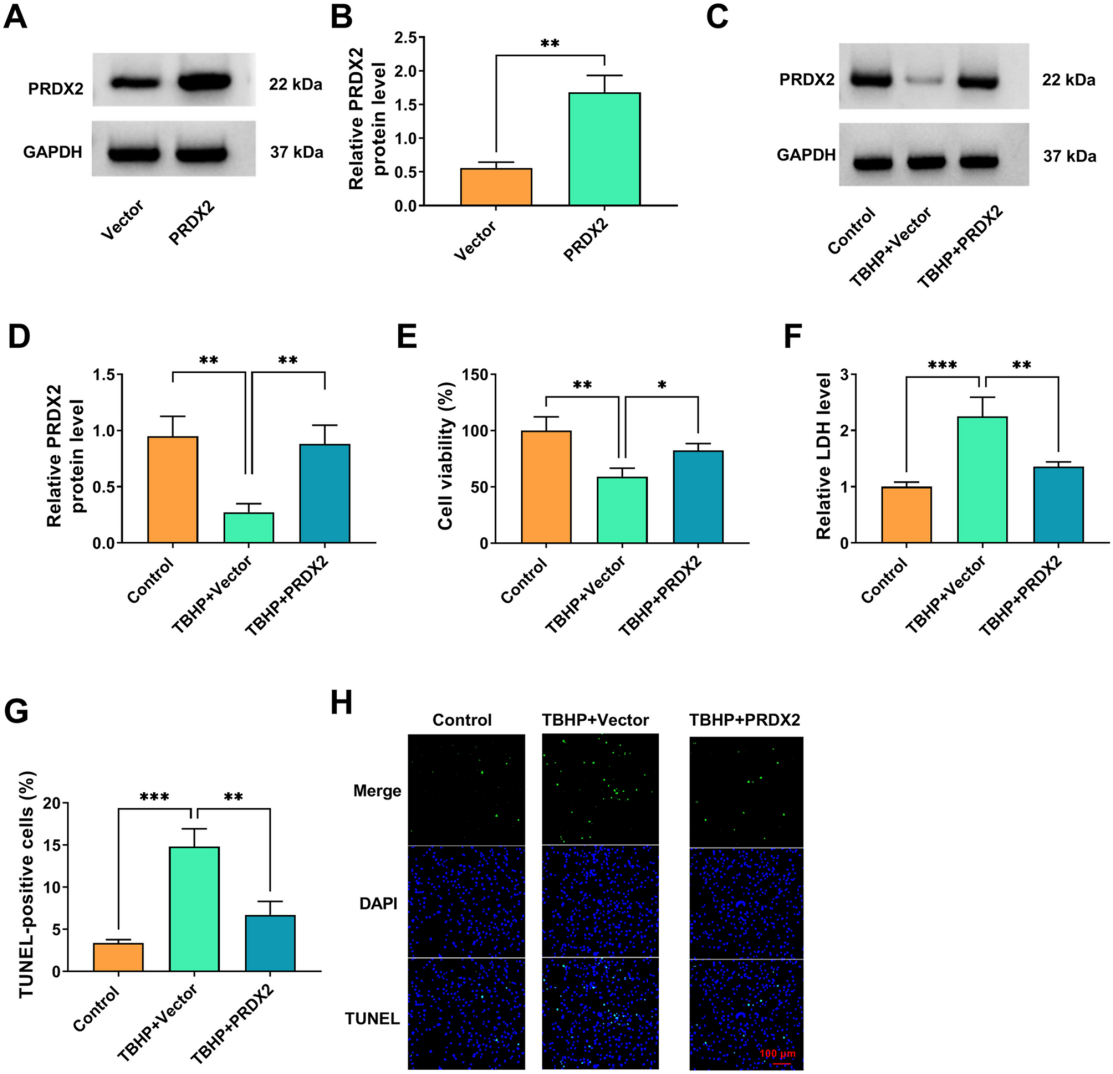
Effect of PRDX2 on TBHP-induced NP cell apoptosis. (**A-B**) The transfection efficiency of PRDX2 overexpression vector was confirmed by WB (*n* = 3). (**C-H**) NP cells were transfected with vector/PRDX2 and treated with 100 µM TBHP for 3 h (*n* = 3). (**C-D**) PRDX2 protein level was tested by WB. (**E**) Cell viability was detected by CCK8 assay. (**F**) LDH level was examined to assess the cytotoxicity of cells. (**G-H**) Cell apoptosis was evaluated using TUNEL staining (100×). The bar plot in G is based on the analysis of the images in H. **A**, Student’s *t*-test; **D, E, F** and **G**, one-way ANOVA. *P < 0.05, **P < 0.01, ***P < 0.001

To explore the role of PRDX2 in IDD progression, our study performed gain-of-functional experiments. The transfection of PRDX2 overexpression vector markedly promoted PRDX2 protein expression in NP cells (Fig. 2A-B). Then, transfected NP cells were treated with TBHP to mimic IDD conditions. The decreasing effect of TBHP on PRDX2 expression could be abolished by PRDX2 overexpression vector (Fig. 2C-D). Then, our study assessed the effect of PRDX2 overexpression on TBHP-induced NP cell viability and apoptosis. The results showed that TBHP treatment reduced NP cell viability and increased LDH level, while PRDX2 overexpression eliminated these effect (Fig. 2E-F). Also, PRDX2 overexpression reduced TUNEL-positive cells in TBHP-induced NP cells (Fig. 2G-H). Taken together, the gain-of-function assay confirmed that restoring PRDX2 expression could effectively antagonized the tbHP-induced decrease in cell viability and apoptosis in NP cells. This directly demonstrates that PRDX2 plays a key protective role in maintaining NP cell survival and that its downregulation may directly contribute to the abnormal loss of NP cells in IDD.

### PRDX2 reduced ferroptosis and ECM degradation in TBHP-induced NP cells

**Fig. 3:**
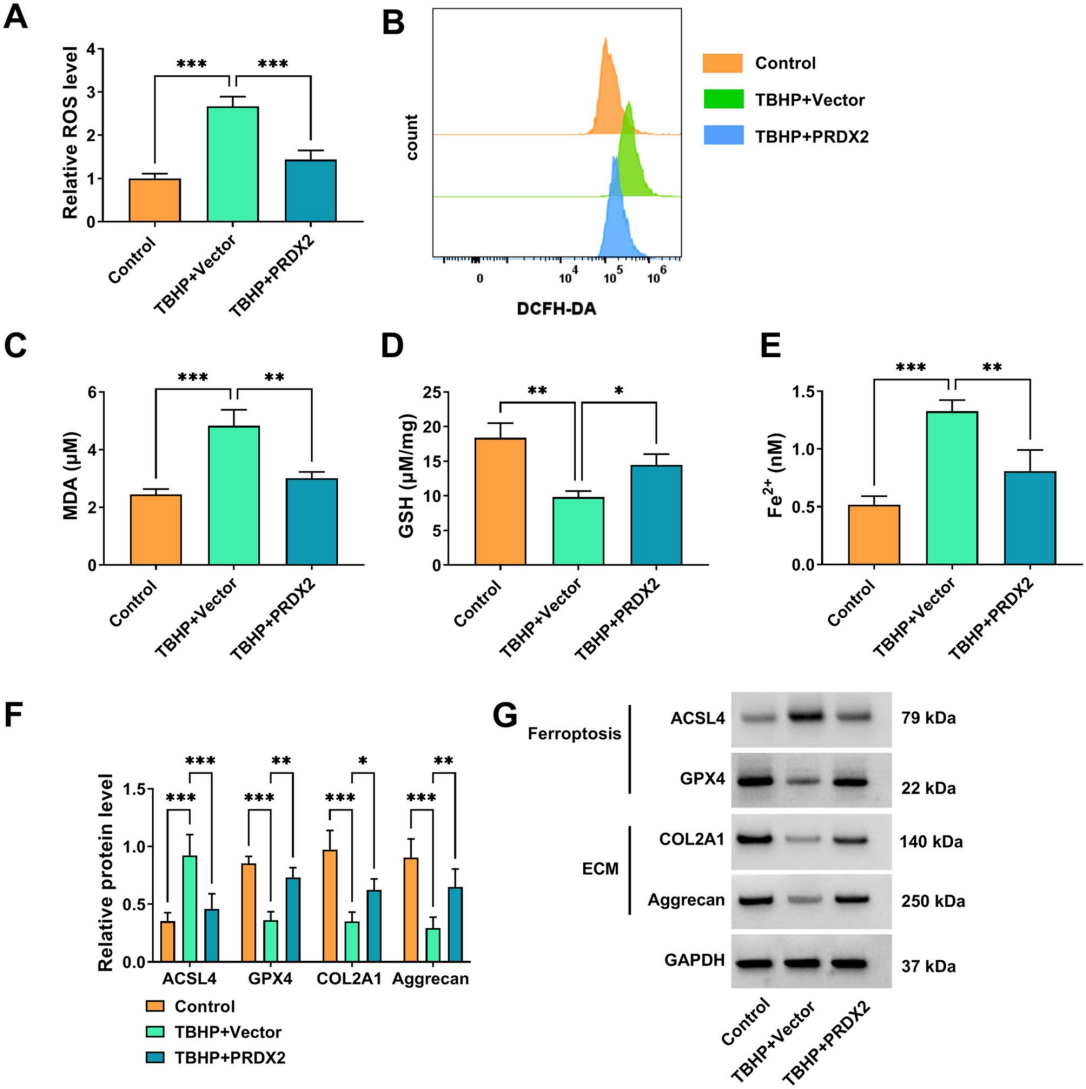
Effect of PRDX2 on TBHP-induced NP cell ferroptosis and ECM degradation. NP cells were transfected with vector/PRDX2 and treated with 100 µM TBHP for 3 h (*n* = 3). (**A-B**) ROS level was examined by DCFDA/H2DCFDA Cellular ROS Assay Kit. DCFHDA is a commonly used fluorescent probe for detecting ROS in cells. The bar plot in **A** is based on the analysis of the DCFH-DA signal in B. (**C-E**) MDA, GSH and Fe^2+^ levels were detected to assess cell ferroptosis. (**F-G**) ACSL4, GPX4, COL2A1 and Aggrecan protein levels were examined by WB. **A**, **C**, **D** and **E**, one-way ANOVA; F, two-way ANOVA. *P < 0.05, **P < 0.01, ***P < 0.001

Following, our study measured the effect of PRDX2 on TBHP-induced NP cell ferroptosis and ECM degradation. TBHP treatment promoted ROS, MDA, and Fe^2+^ levels, while decreased GSH level in NP cells. However, these effects were reversed by PRDX2 overexpression (Fig. 3A-E). Besides, our study detected the protein levels of ferroptosis-related markers (ACSL4 and GPX4) and ECM-related markers (COL2A1 and Aggrecan). The results indicated that PRDX2 decreased ACSL4 level, while enhanced GPX4, COL2A1 and Aggrecan levels in TBHP-induced NP cells (Fig. 3F-G). Therefore, PRDX2 not only inhibits apoptosis, but also comprehensively resists TBHP-induced ferroptosis by regulating REDOX homeostasis (decreasing ROS/MDA, increasing GSH) and iron metabolism (decreasing Fe^2+^), and affecting key proteins (such as ACSL4, GPX4). At the same time, PRDX2 can effectively maintain the synthesis of the main components of ECM (COL2A1 and Aggrecan), and protect the function and microenvironment homeostasis of NP cells from multiple levels.

### USP11 promoted the stability of PRDX2 protein through deubiquitination

**Fig. 4:**
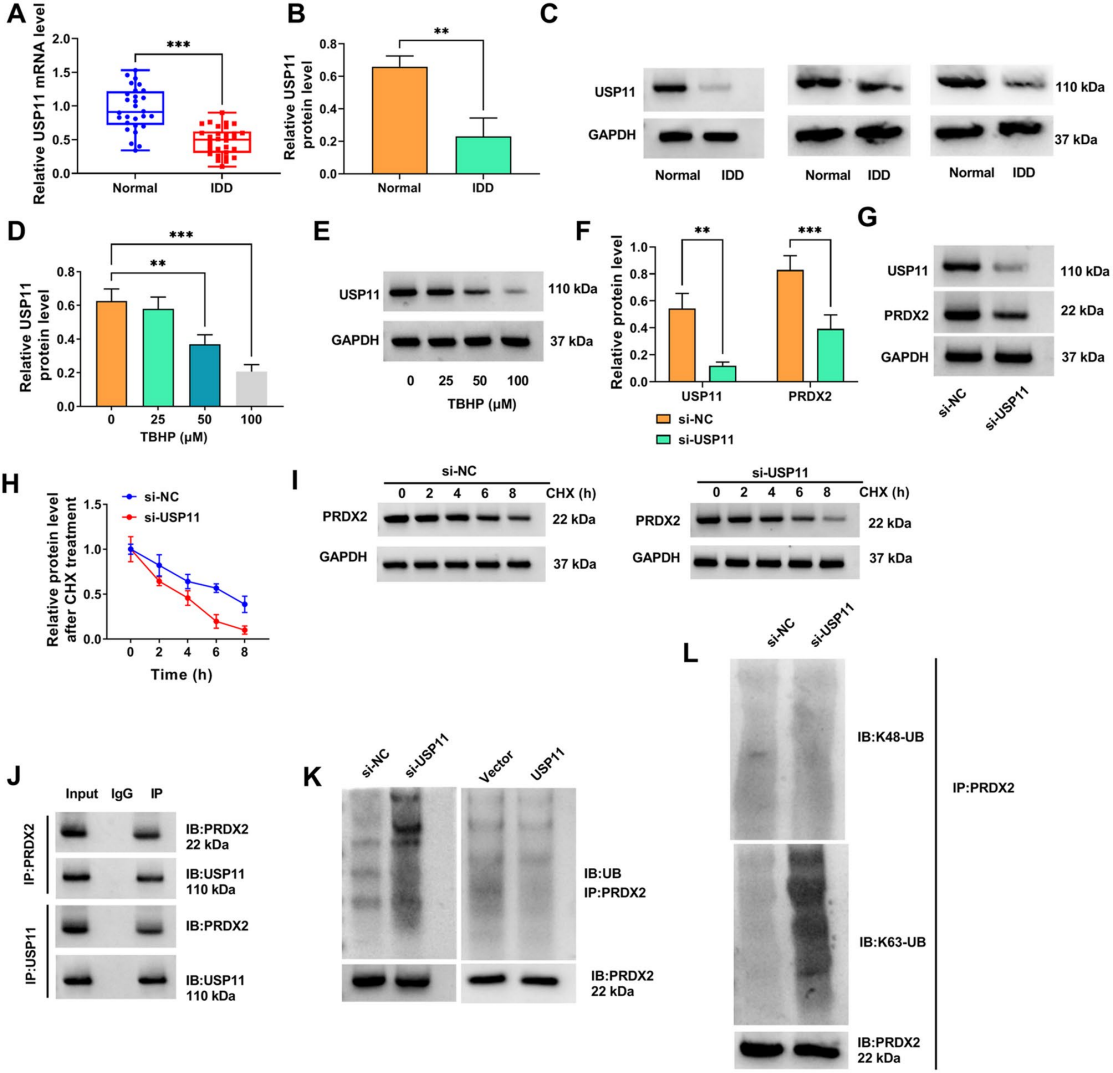
USP11 regulated PRDX2 through deubiquitination. (**A**) USP11 mRNA level in the NP tissues of IDD patients (*n* = 30) and normal controls (*n* = 28) was measured by qRT-PCR. The dots represent individual patient samples. (**B-C**) USP11 protein level was detected by WB in the NP tissues of IDD patients and normal controls. Data were derived from 3 independent human tissue samples (*n* = 3). (**D-E**) USP11 protein level in NP cells treated with different concentrations of TBHP for 3 h was examined by WB (*n* = 3). (**F-G**) USP11 and PRDX2 protein levels in NP cells transfected with si-NC/si-USP11 were detected by WB (*n* = 3). (**H-I**) CHX treatment assay was used to assess the stability of PRDX2 protein in NP cells transfected with si-NC/si-USP11 (*n* = 3). Co-IP assay (**J**) and ubiquitination assay (**K**) were used to detect the interaction between PRDX2 and USP11, as well as the effect of USP11 knockdown or overexpression on PRDX2 ubiquitination. (**L**) Ubiquitination assay was used to measure the effect of si-USP11 on the K48/K63-type ubiquitination modification of PRDX2. **A** and **B**, Student’s *t*-test; **D**, one-way ANOVA; **F** and **H**, two-way ANOVA. **P < 0.01, ***P < 0.001

USP11 expression was lower in the NP tissues of IDD patients than that in normal controls at the mRNA and protein levels (Fig. 4A-C). Furthermore, TBHP treatment significantly reduced USP11 protein expression in NP cells in a concentration dependent manner (Fig. 4D-E). After confirmed that si-USP11 transfection could reduce USP11 protein expression in NP cells, our study found that PRDX2 protein expression was significantly decreased by si-USP11 (Fig. 4F-G). Besides, CHX treatment assay was performed to assess the effect of si-USP11 on PRDX2 protein stability. The results showed that USP11 knockdown inhibited the stability of PRDX2 protein (Fig. 4H-I). Besides, Co-IP assay was carried out to measure the interaction between USP11 and PRDX2, and the data revealed that USP11 protein interacted with PRDX2 protein (Fig. 4J). Moreover, ubiquitination assay suggested that USP11 knockdown promoted PRDX2 ubiquitination, while its overexpression had an opposite effect (Fig. 4K). In addition, K63-type ubiquitination modification on PRDX2 protein was specifically increased after USP11 knockdown, while K48-type modification was not significantly changed (Fig. 4L). These data suggests that USP11 maintains PRDX2 stability by specifically removing the K63-linked ubiquitin chain from PRDX2, revealing a core regulatory mechanism upstream of PRDX2.

### USP11 promoted PRDX2 expression to inhibit TBHP-induced NP cell apoptosis

**Fig. 5:**
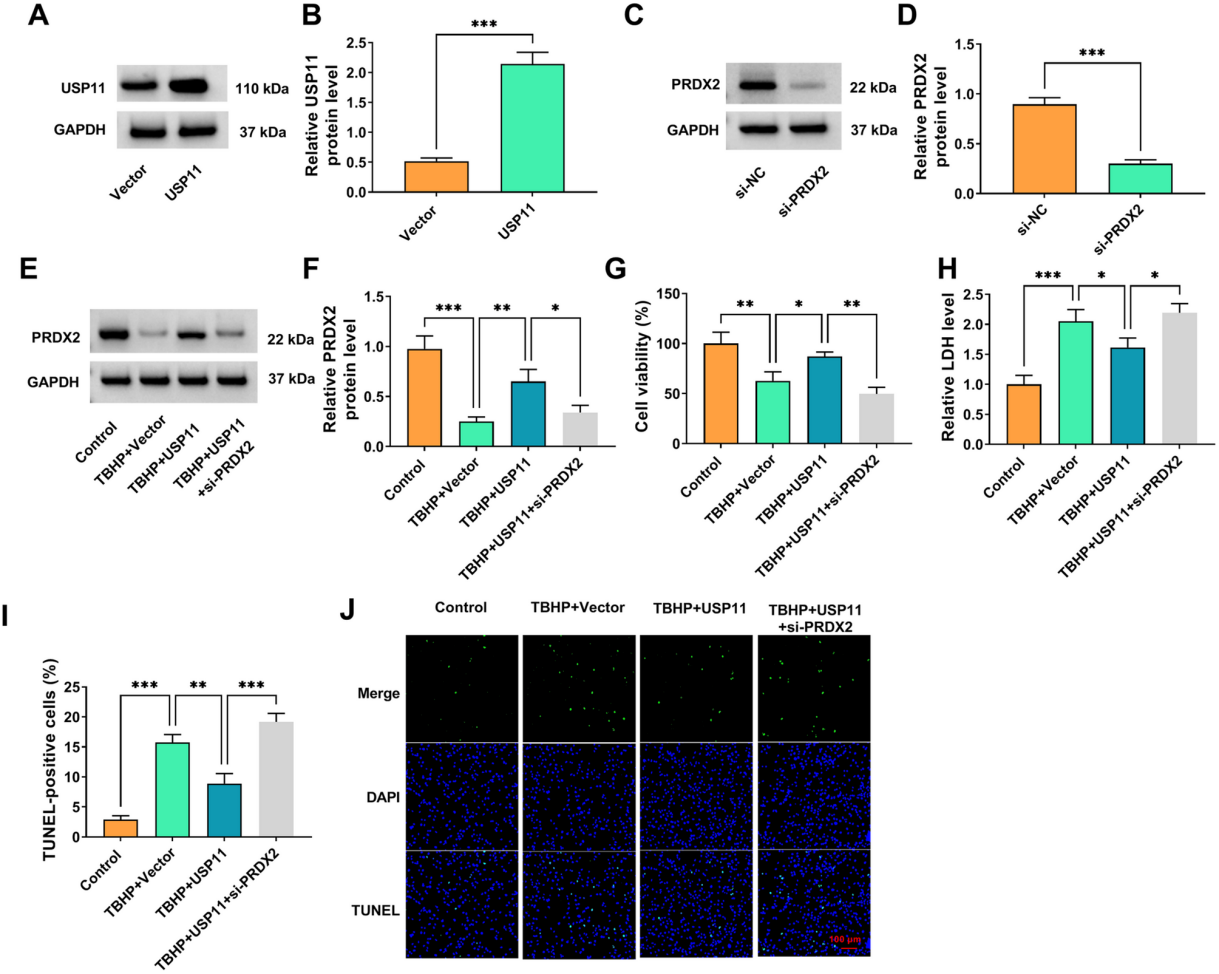
Effect of USP11 and si-PRDX2 on TBHP-induced NP cell apoptosis. (**A-B**) The transfection efficiency of USP11 overexpression vector was confirmed by WB (*n* = 3). (**C-D**) The transfection efficiency of si-PRDX2 was assessed by WB (*n* = 3). (**E-J**) NP cells were transfected with vector/USP11/si-PRDX2 and treated with 100 µM TBHP for 3 h (*n* = 3). (**E-F**) PRDX2 protein level was examined by WB. (**G**) CCK8 assay was used to measure cell viability. (**H**) Cell cytotoxicity was assessed by detecting LDH level. (**I-J**) TUNEL staining was performed to measure cell apoptosis (100×). The bar plot in **I** is based on the analysis of the images in **J**. **B** and **D**, Student’s *t*-test; **F**-**I**, one-way ANOVA. *P < 0.05, **P < 0.01, ***P < 0.001

To further investigate the role of USP11 in IDD progression and whether USP11 regulated PRDX2 to mediate IDD progression, our study performed the rescue experiments. USP11 protein expression could be promoted by USP11 overexpression vector (Fig. 5A-B), and si-PRDX2 was used to reduce PRDX2 protein expression (Fig. 5C-D). In TBHP-induced NP cells transfected with USP11 overexpression vector and si-PRDX2, our study confirmed that si-PRDX2 reversed the increasing effect of USP11 on PRDX2 protein level (Fig. 5E-F). Meanwhile, USP11 overexpression enhanced viability, while reduced LDH level and TUNEL-positive cells in TBHP-induced NP cells. However, PRDX2 knockdown overturned these effects (Fig. 5G-J). The results of the rescue experiments establish a clear downstream causal relationship between USP11 and PRDX2. The anti-apoptotic protective effect of USP11 overexpression was completely reversed by PRDX2 knockdown. This strongly suggests that USP11 exerts its protective effect primarily by maintaining the protein level of PRDX2.

### USP11 repressed TBHP-induced NP cell ferroptosis and ECM degradation by regulating PRDX2

**Fig. 6:**
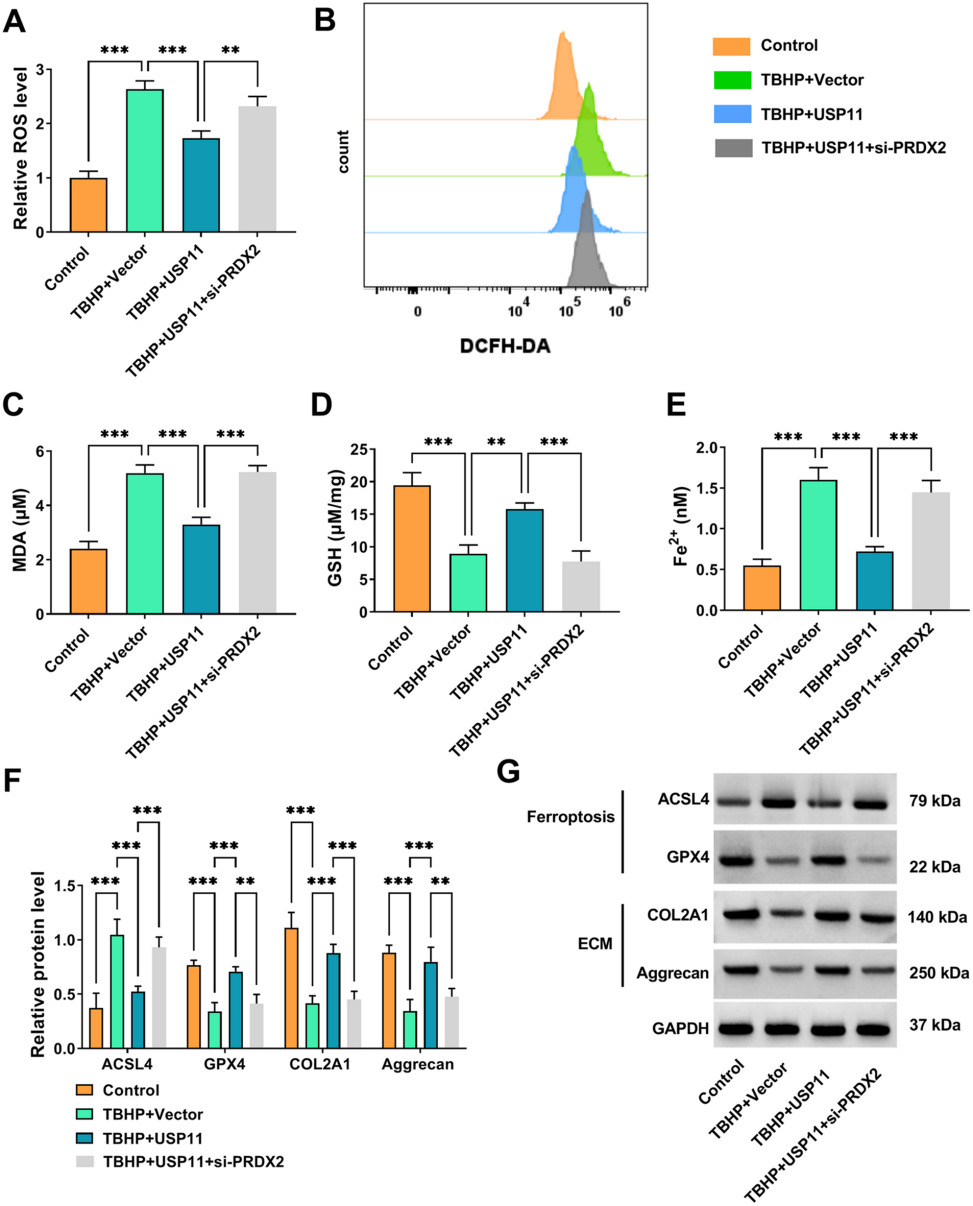
Effect of USP11 and si-PRDX2 on TBHP-induced NP cell ferroptosis and ECM degradation. NP cells were transfected with vector/USP11/si-PRDX2 and treated with 100 µM TBHP for 3 h (*n* = 3). (**A-B**) ROS level was examined using DCFDA/H2DCFDA Cellular ROS Assay Kit. DCFHDA is a commonly used fluorescent probe for detecting ROS in cells. The bar plot in **A** is based on the analysis of the DCFH-DA signal in **B**. (**C-E**) Cell ferroptosis was assessed to measure MDA, GSH and Fe^2+^ levels. (**F-G**) WB was used to test ACSL4, GPX4, COL2A1 and Aggrecan protein levels. **A**, **C**,** D** and **E**, one-way ANOVA; F, two-way ANOVA. **P < 0.01, ***P < 0.001

Moreover, our study assessed the effect of USP11 overexpression and si-PRDX2 on the ferroptosis and ECM degradation in TBHP-induced NP cells. The results indicated that USP11 overexpression inhibited ROS, MDA, Fe^2+^ and promoted GSH levels in TBHP-induced NP cells, while PRDX2 knockdown abolished these effects (Fig. 6A-E). WB analysis revealed that upregulation of USP11 decreased ACSL4 level, increased GPX4, COL2A1 and Aggrecan levels in TBHP-induced NP cells, whereas these effects could be eliminated by downregulating PRDX2 (Fig. 6F-G). This finally validated our proposed core axis: under pathological stress of IDD, USP11 stabilizes PRDX2 through deubiquitination, which mediates multiple protective responses against apoptosis, ferroptosis and ECM degradation. Failure of this regulatory axis may become an important driving factor in the progression of IDD.

Supplementary files The full length original WB images.

## Discussion

The main clinical manifestations of IDD are lumbar pain, stiffness and lower limb numbness, which seriously affect the quality of life of patients [19, 20]. Dysfunction of NP cells is one of the main causes of IDD [21], so it is important to clarify the molecular mechanisms affecting NP cell functions. In the present study, our study used TBHP to induce an IDD environment and determined that TBHP could inhibit NP cell viability and promote apoptosis, ferroptosis, and ECM degradation, which was consistent with previous reports [22, 23].

PRDX2 plays a vital role in fighting ROS and regulating cell growth through its peroxidase function. PRDX2 mediated atherosclerosis process, which decreased ROS level, promoted proliferation and enhanced migration in carotid artery vascular smooth muscle cells [24]. Moreover, PRDX2 inhibited oxidative stress and apoptosis in myocardial tissues of rats by reducing ROS level and caspase-related protein levels, thus alleviating myocardial injury caused by acute myocardial infarction [25]. Thus, aberrant PRDX2 expression is closely related to human disease progression. Although Tu et al. [10] revealed the low expression of PRDX2 in NP tissues, its role in IDD progression has not been explored. In this, our study detected the downregulated PRDX2 level in IDD patients and TBHP-induced NP cells. Besides, gain-of-functional experiments revealed that upregulation of PRDX2 enhanced viability, while suppressed apoptosis, ferroptosis and ECM degradation in TBHP-induced NP cells, which was consistent with previous reports that PRDX2 had anti-apoptotic, anti-ferroptosis and anti-ECM degradation effects [8, 9]. These findings suggested that PRDX2 might alleviate IDD progression by inhibiting TBHP-induced NP cell dysfunction.

Ubiquitination is a post-translational modification of proteins that is accomplished by a series of enzymatic reactions [26]. Deubiquitinating enzymes are responsible for the cleavage of ubiquitin from target proteins, and then participate in regulating protein stability and expression [27]. Targeting deubiquitinating enzymes may be a potential therapeutic strategy for many diseases [28]. As a deubiquitinating enzyme, the function of USP11 has been extensively explored. USP11 accelerated spinal cord ischemia-reperfusion injury, which increased autophagy-dependent ferroptosis in neuronal cells by deubiquitinating Beclin1 [29]. USP11 deubiquitinated IGF2BP3 to promote its expression, thus accelerating colorectal cancer cell proliferation and metastasis [30]. Besides, USP11 is verified to have anti-apoptosis and anti-ferroptosis roles in many types of cells [17, 31, 32]. In this, our study detected that USP11 was lowly expressed in IDD patients. Consistent with the results of Zhu et al. [18], our study confirmed the inhibitory effect of USP11 on TBHP-induced NP cell ferroptosis. Moreover, our study also revealed that USP11 could inhibit apoptosis and ECM degradation in TBHP-induced NP cells. In a novel finding, our study determined that USP11 could stabilize PRDX2 protein expression by deubiquitination, and further analysis suggested that USP11-mediated anti-apoptotic, anti-ferroptosis and anti-ECM degradation in TBHP-induced NP cells could be reversed by PRDX2 knockdown. These results further confirm the conclusion that USP11-regulated PRDX2 alleviates IDD progression.

Of course, there are still some limitations of this study. The main findings of the present study are all based on the TBHP-induced NP cell degeneration model. Although TBHP is a widely recognized and used oxidative stress inducer, which can effectively simulate the key pathological process of IDD, the complex microenvironment, cell-cell interactions and systemic feedback in vivo cannot be fully recapitalized in cell models. Future studies will be devoted to the establishment of animal models of IDD (such as rat acupuncture model or mouse spontaneous degeneration model) to verify the function and intervention effect of PRDX2 regulation at the in vivo level. Moreover, our study were not able to retrospectively assess the Pfirrmann grade for all enrolled cases or provide the results of histologic staining such as safranin-O fast green. This is mainly due to the limitation of retrospective studies (it is not possible to obtain the original MRI images of all patients for uniform grading) and the limitation of sample resources (some samples have been completely used for molecular biology experiments and can no longer be prepared for staining). In the future, samples will need to be collected to refine the results. In addition, the evidence supporting ferroptosis in this study is currently limited to ROS, GSH, Fe^2+^, MDA, and ACSL4/GPX4, and fails to provide direct evidence of mitochondrial morphology by transmission electron microscopy. Future studies are needed to further confirm the occurrence of ferroptosis by ultrastructural observation in animal models or in vitro models that retain better cellular architecture.

To sum up, our study reveals a novel molecular mechanism regulating IDD progression. PRDX2, deubiquitinated by USP11, restrained TBHP-induced NP cell apoptosis, ferroptosis and ECM degradation. This provides a new theoretical basis for developing the potential molecular targets to alleviate the IDD process.

## Supplementary Information

Below is the link to the electronic supplementary material.

Supplementary Material 1

## Data Availability

Not applicable.
