# Supplementary material for: Ubiquitination-mediated PRDX2 alleviates intervertebral disc degeneration via restraining TBHP-induced nucleus pulposus cell apoptosis, ferroptosis and ECM degradation
Source: J Inflamm (Lond). 2026 Mar 24;23:15. doi: 10.1186/s12950-026-00493-x (PMC13134154; doi:10.1186/s12950-026-00493-x)

Fig 1C

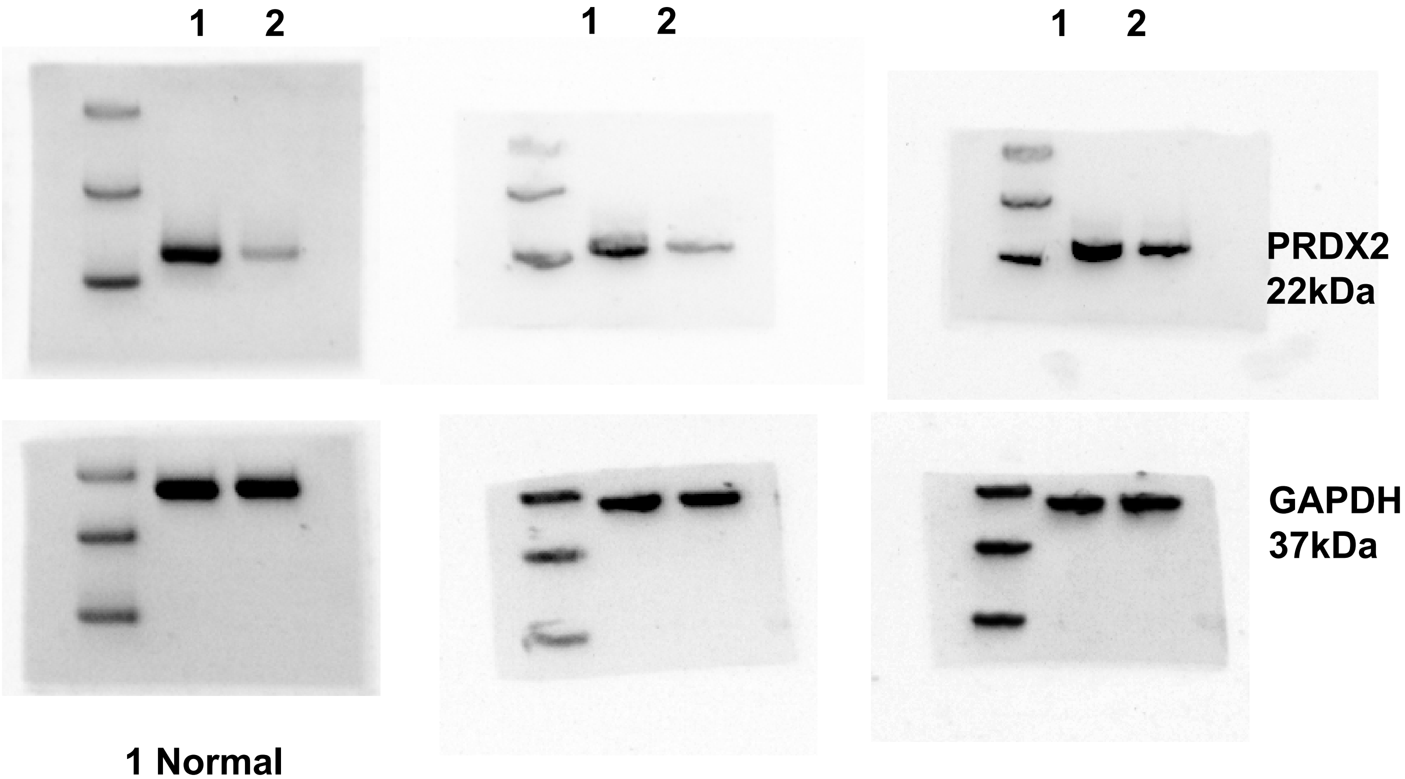

1 Normal

2 IDD

Fig 1E

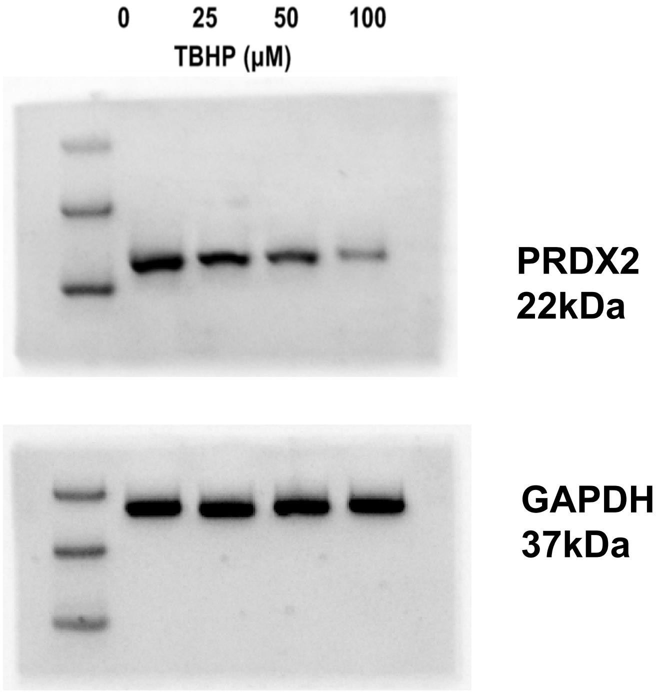

Fig 2A

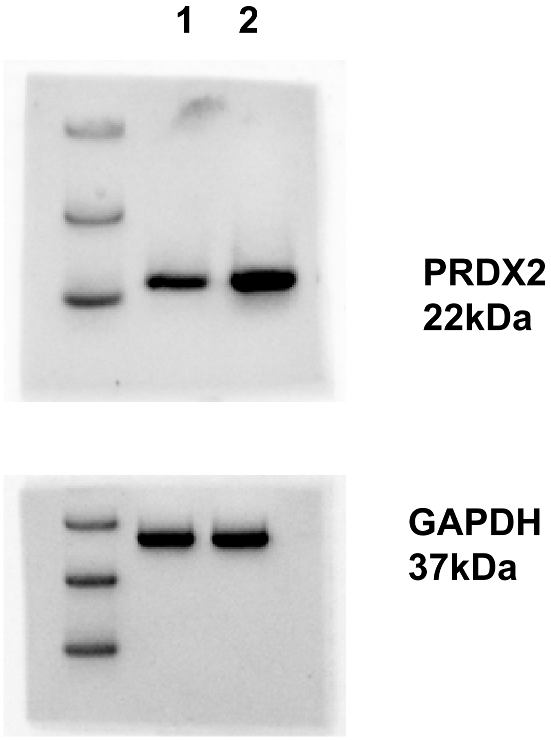

1 vector

2 PRDX2

Fig 2C

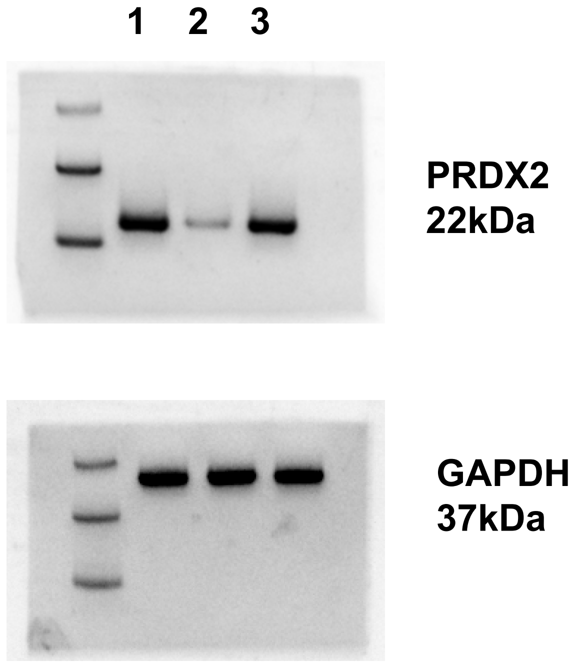

1 Control

2 TBHP+vector

3 TBHP+PRDX2

Fig 3G

1 Control

2 TBHP+vector

3 TBHP+PRDX2

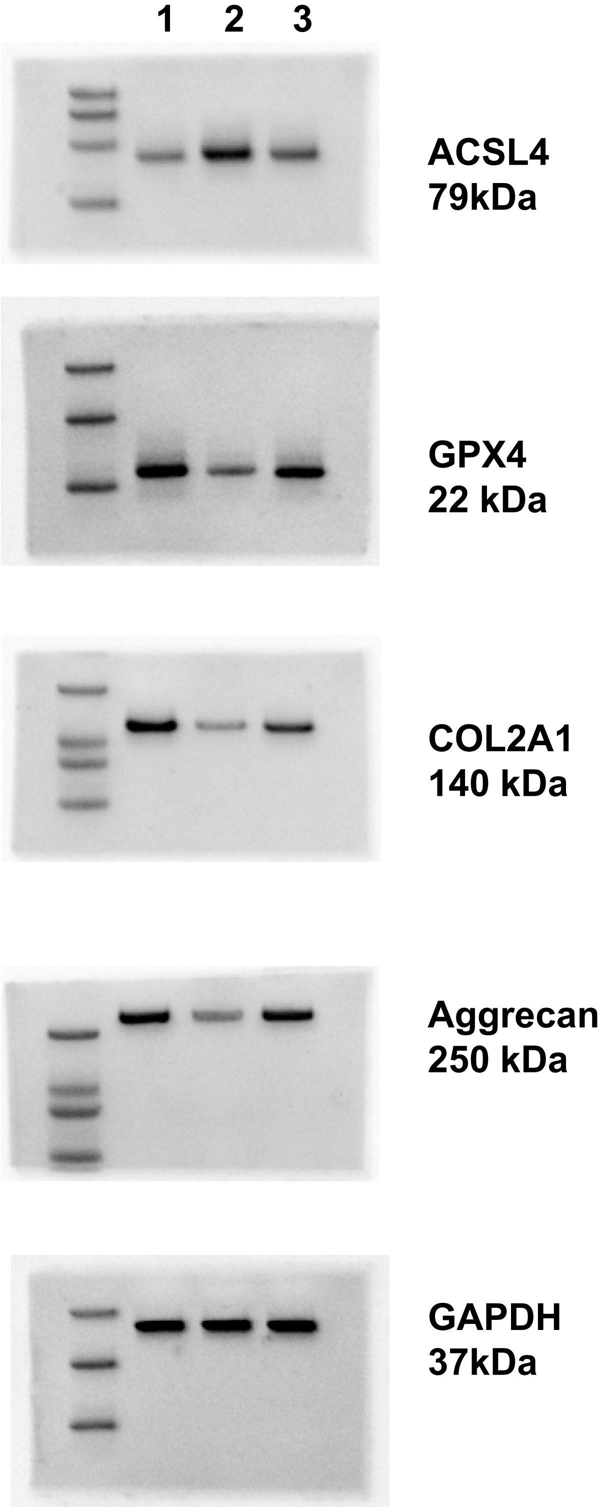

Fig 4C

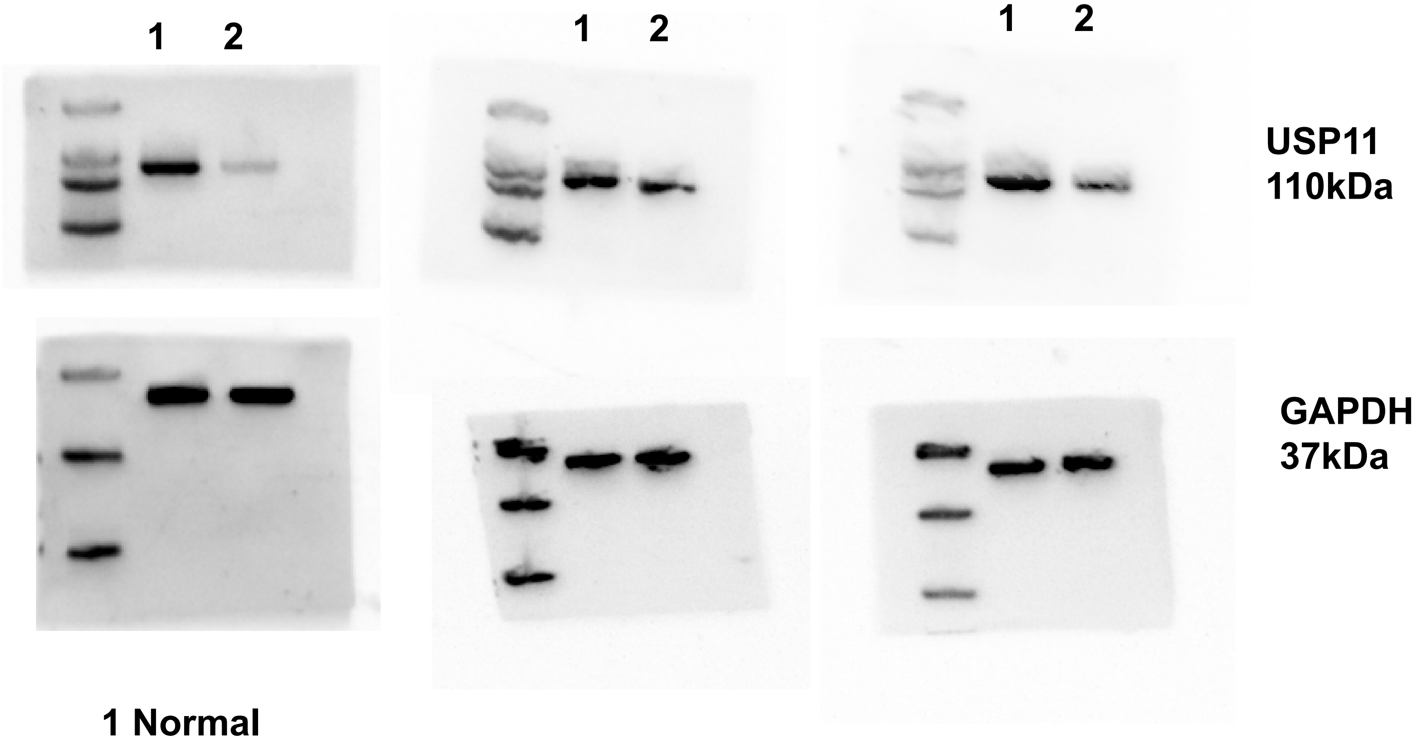

1 Normal

2 IDD

Fig 4E

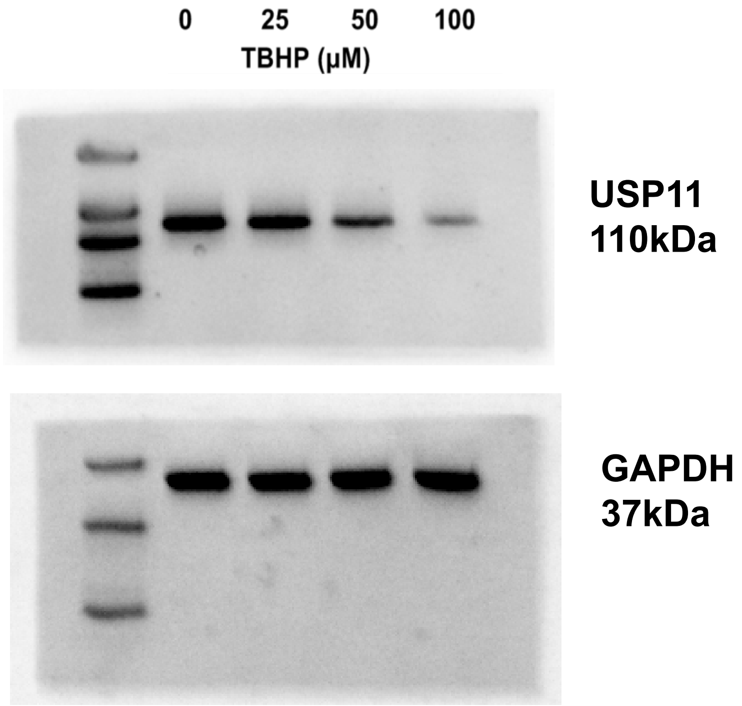

0
25
50
100
TBHP (μM)
USP11
110kDa
GAPDH
37kDa

Fig 4G

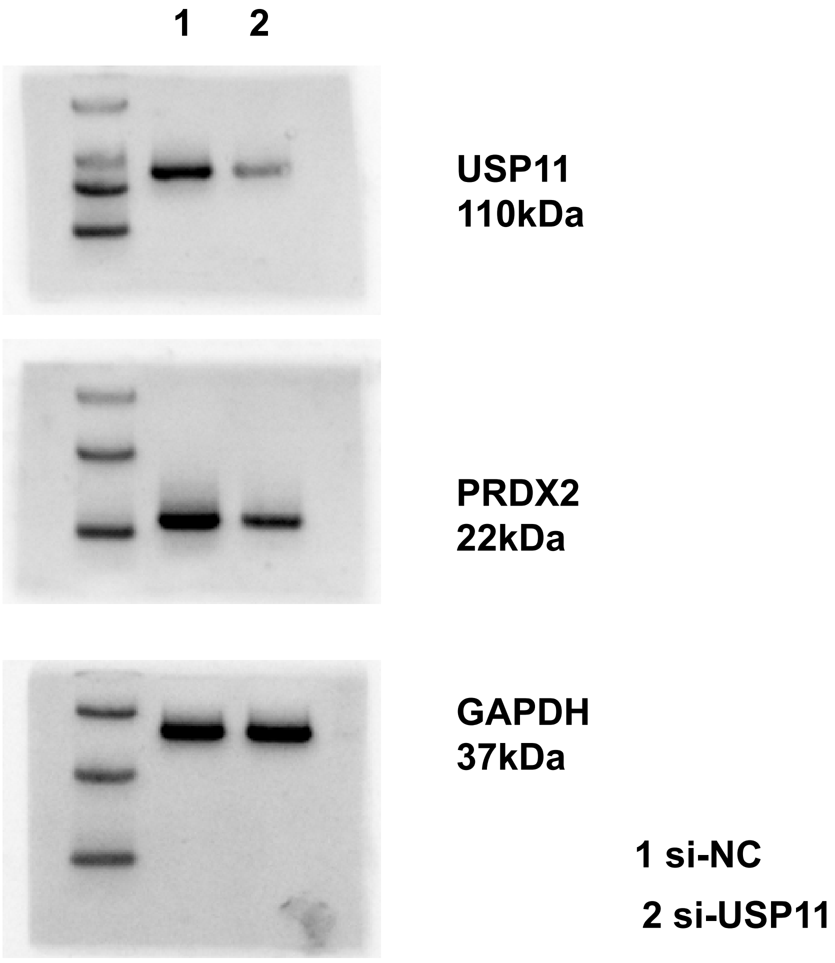

1
2
USP11
110kDa
PRDX2
22kDa
GAPDH
37kDa
1 si-NC
2 si-USP11

Fig 4l

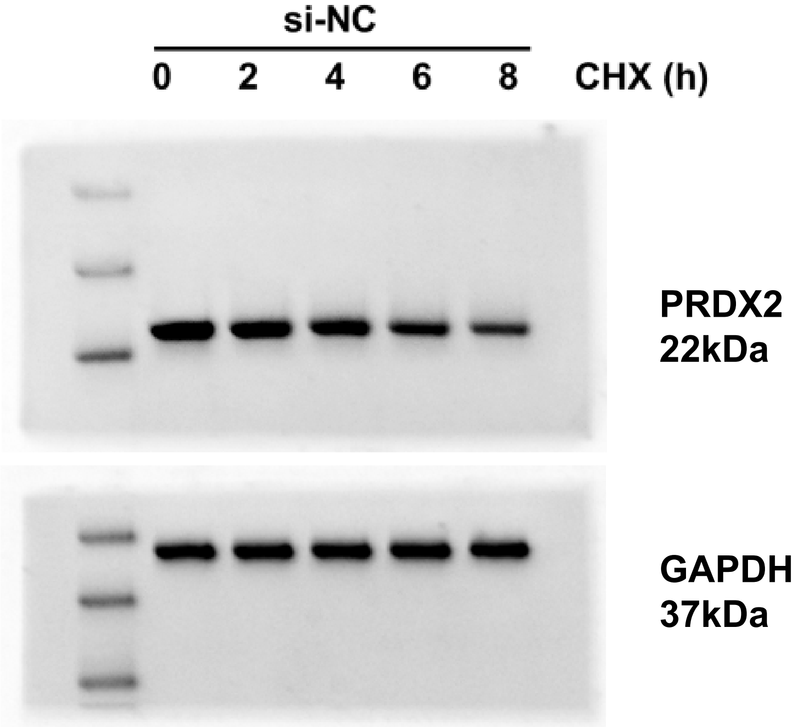
si-NC
0 2 4 6 8 CHX (h)
PRDX2
22kDa
GAPDH
37kDa

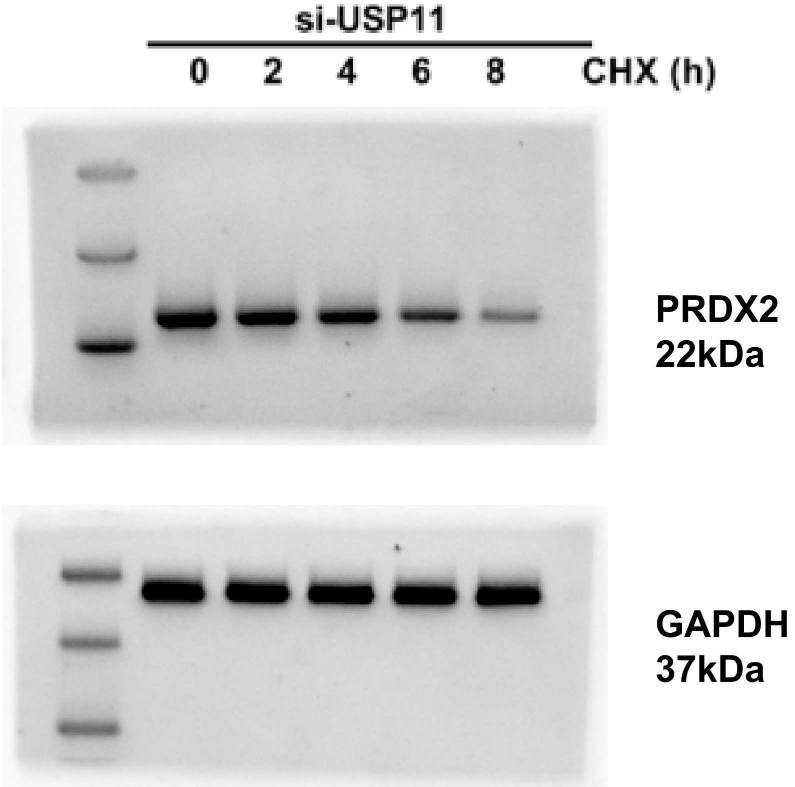
si-USP11
0 2 4 6 8 CHX (h)
PRDX2
22kDa
GAPDH
37kDa

Fig 4J

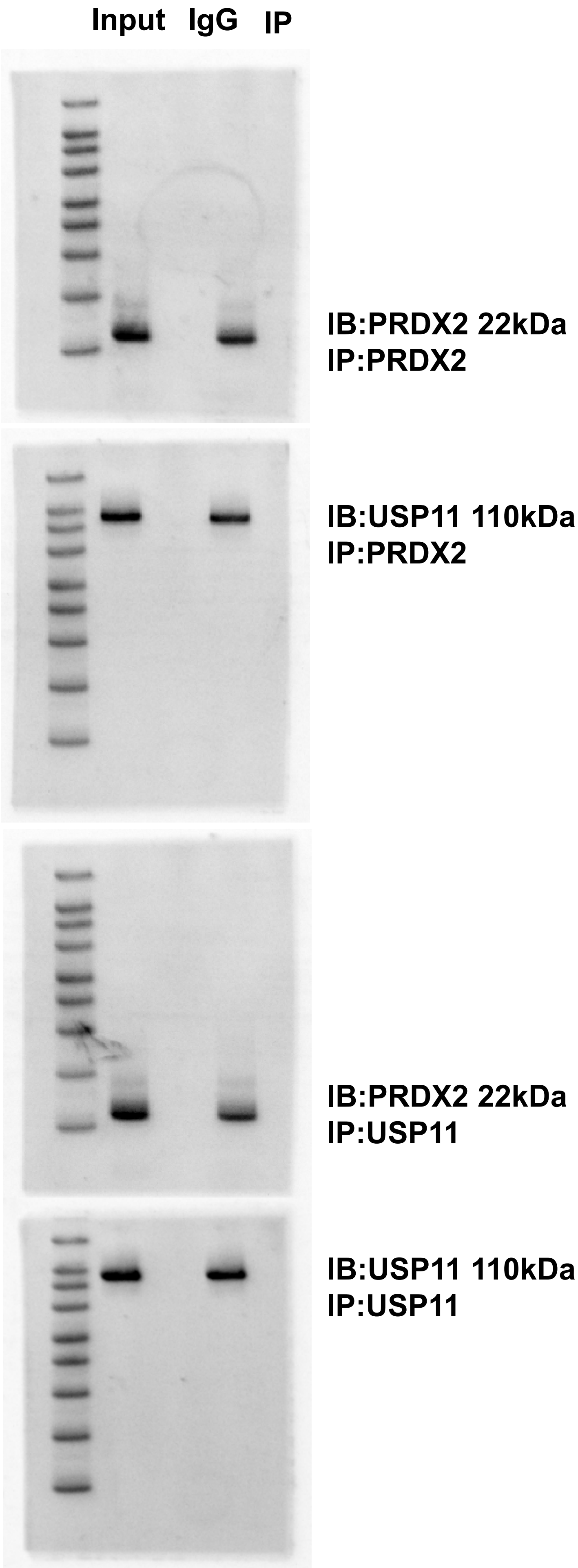
Input
IgG
IP
IB:PRDX2 22kDa
IP:PRDX2
IB:USP11 110kDa
IP:PRDX2
IB:PRDX2 22kDa
IP:USP11
IB:USP11 110kDa
IP:USP11

Fig 4K 1 si-NC

2 si-USP11

1 2

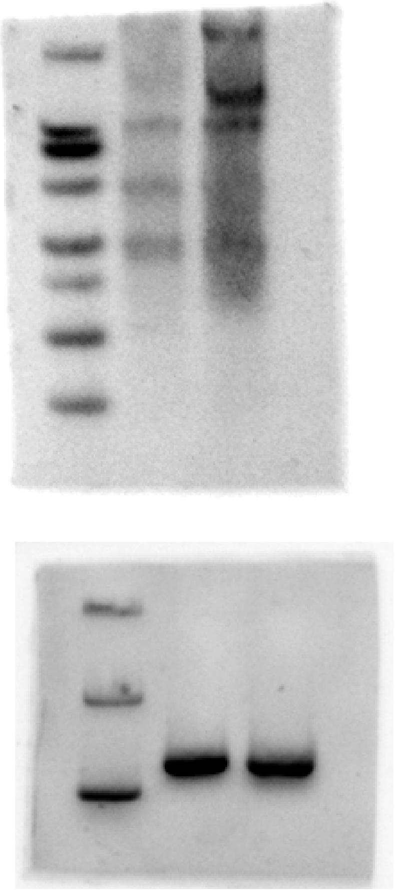

IB:UB
IP:PRDX2

IB:PRDX2 22 kDa
IP:PRDX2

Fig 4L

1 si-NC

2 si-USP11

1 2

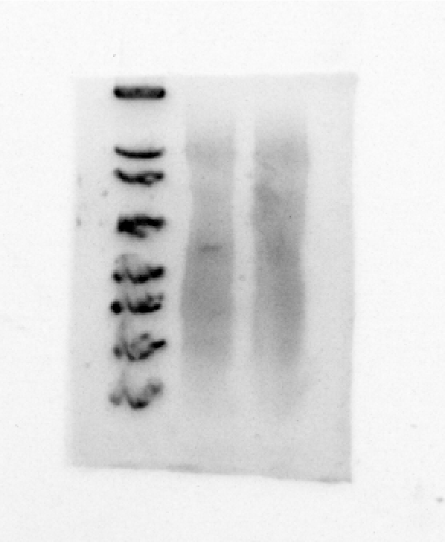

IB:K48-UB
IP:PRDX2

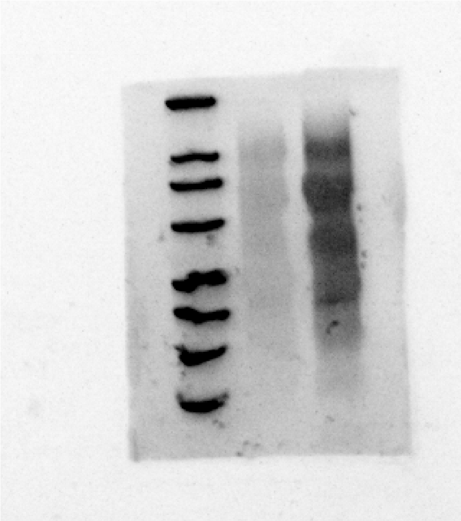

IB:K63-UB
IP:PRDX2

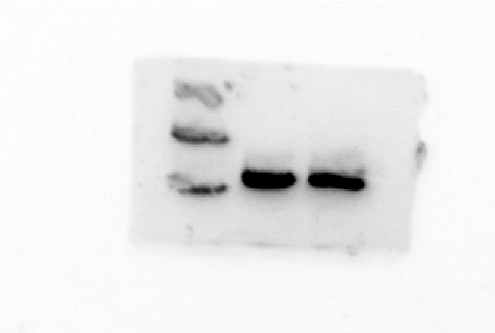

IB:PRDX2 22 kDa
IP:PRDX2

Fig 5A

1 vector

2 USP11

1 2

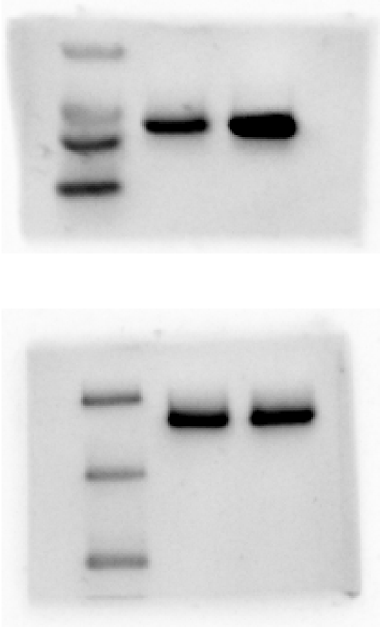

USP11
110kDa

GAPDH
37kDa

Fig 5C

1 si-NC

2 si-PRDX2

1 2

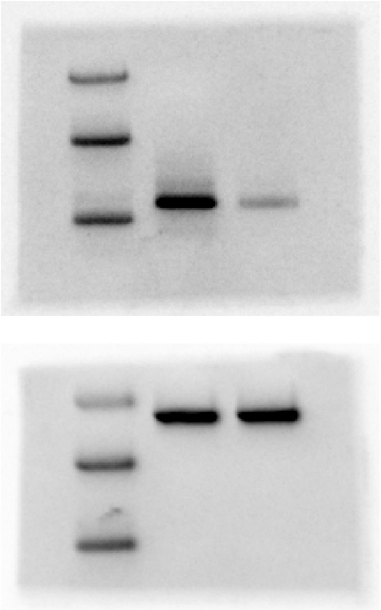

PRDX2
22kDa

GAPDH
37kDa

Fig 5E

1 2 3 4

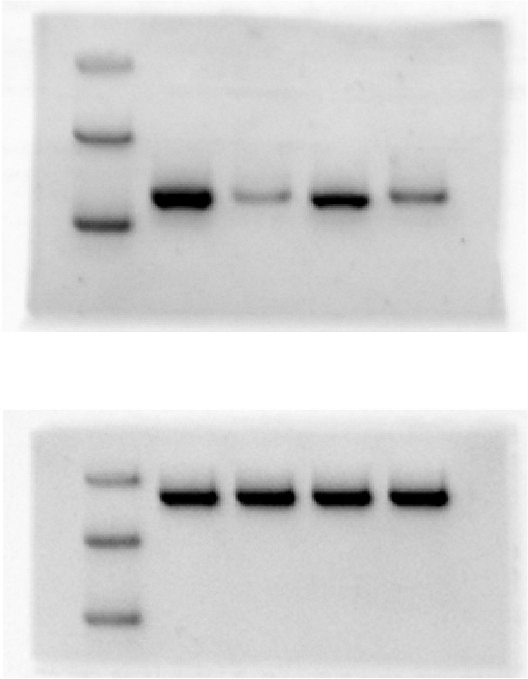

PRDX2
22kDa

GAPDH
37kDa

1 Control

2 TBHP+vector

3 TBHP+USP11

4 TBHP+USP11+si-PRDX2

Fig 6G

1 Control

2 TBHP+vector

3 TBHP+USP11

4 TBHP+USP11+si-PRDX2

1 2 3 4

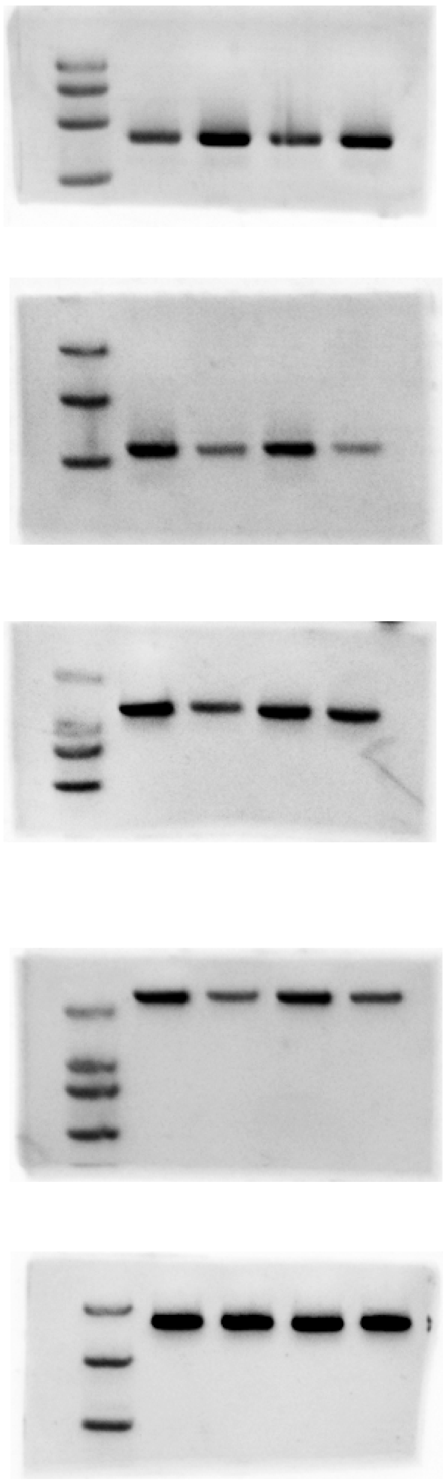

ACSL4
79kDa

GPX4
22 kDa

COL2A1
140 kDa

Aggrecan
250 kDa

GAPDH
37kDa

- **The samples derive from the same experiment and that gels/blots were processed in parallel.**

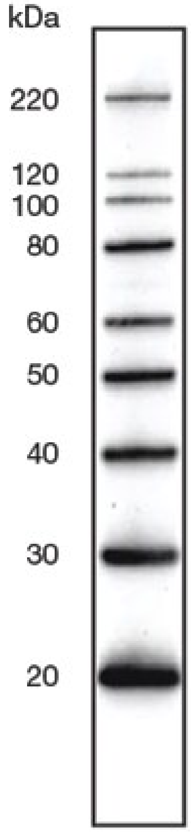

Supplement: Supplementary file 1 — Supplementary Material 1 [file 12950_2026_493_MOESM1_ESM.pdf]
